# Epicardial fat density obtained with computed tomography imaging - more important than volume?

**DOI:** 10.1186/s12933-024-02474-x

**Published:** 2024-10-29

**Authors:** Łukasz Nogajski, Maciej Mazuruk, Marta Kacperska, Mikołaj Kurpias, Maciej Mączewski, Maksymilian Nowakowski, Michał Mączewski, Ilona Michałowska, Przemysław Leszek, Aleksandra Paterek

**Affiliations:** 1 Department of Clinical Physiology, Centre of Postgraduate Medical Education, Warsaw, Poland; 2 Student’s Cardiovascular Scientific Club “Kardioplegia”, Medical University of Warsaw, Warsaw, Poland; 3 Department of Radiology, National Institute of Cardiology, Warsaw, Poland; 4 Heart Failure and Transplantology Department, Mechanical Circulatory Support and Transplant Department, National Institute of Cardiology, Warsaw, Poland

**Keywords:** Epicardial fat, Coronary artery disease, Atherosclerosis, Inflammation, Atrial fibrillation, Heart failure

## Abstract

Epicardial adipose tissue (EAT) is a unique fat depot located between the myocardium and the visceral layer of pericardium. It can be further subdivided into pericoronary (PCAT), periatrial (PAAT) and periventricular adipose tissue (PVentAT), each of them exhibiting specific characteristics and association with the underlying tissue. Since no physical barrier separates EAT from the myocardium, this fat tissue can easily interact with the underlying cardiac structure. EAT can be visualized using various imaging modalities. Computed tomography provides not only information on EAT volume, but also on its density. Indeed, EAT density reflected by the recently developed fat attenuation index (FAI) is emerging as a useful index of PCAT inflammation, PAAT inflammation and fibrosis, while the relevance of density of PVentAT is much less known. The emerging data indicates that FAI can be an important diagnostic and prognostic tool in both coronary artery disease and atrial fibrillation. Future studies will demonstrate if it also could be used as a marker of efficacy of therapies and whether FAI PVentAT could indicate ventricular pathologies, such as heart failure. The aim of the review is to present computed tomography derived FAI as an important tool both to study and better understand the epicardial fat and as a potential predictive marker in cardiovascular disorders.

## Highlights

- Epicardial fat (EAT) is located between the myocardium and the pericardium.
- EAT density, reflected by fat attenuation index (FAI), reflects its characteristics.
- FAI is emerging as an important diagnostic and prognostic tool in cardiology.

## Epicardial fat - introduction

**Fig. 1:**
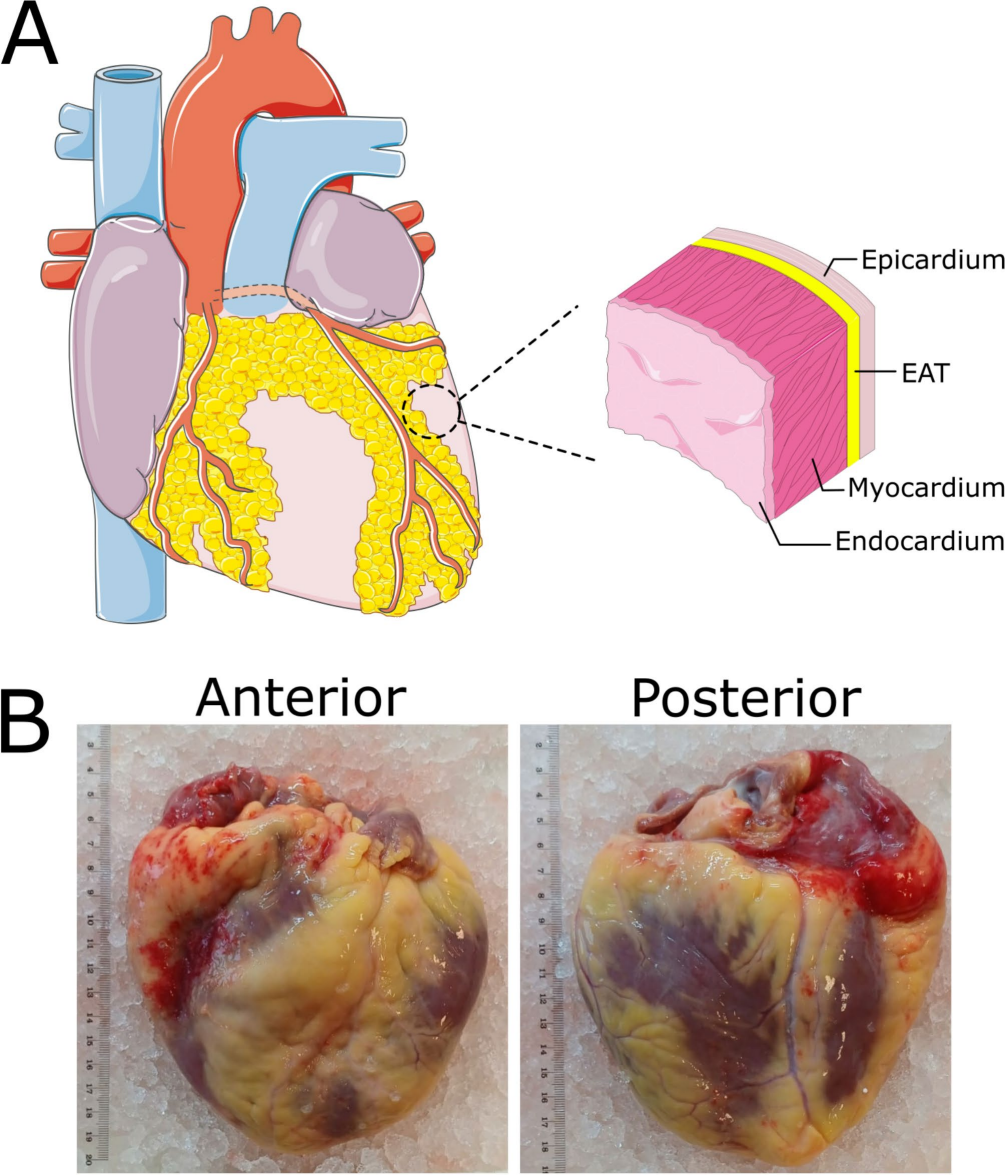
Epicardial fat. [**A**] Schematic representation of epicardial adipose tissue. EAT predominantly surrounds coronary blood vessels and is situated between the myocaridum and the visceral layer of the pericardium. [**B**] Real image of the healthy human heart (our unpublished data) showing the distribution of EAT around the epicardial blood vessels, particularly in the atrioventricular and interventricular sulci, with less fat observed on the posterior side

Epicardial adipose tissue (EAT) is a unique fat depot located between the myocardium and the visceral layer of pericardium (Fig. 1). On the other hand, the pericardial fat is located between the visceral and parietal pericardium, while paracardial fat is located superior to the visceral pericardium and around large blood vessels [1, 2].

As any adipose tissue it is composed essentially of adipocytes containing predominantly lipid droplets. It not only stores fat as an energy source, but also is a producer of multiple bioactive molecules. Since no physical barrier separates EAT from the myocardium and EAT adheres to the cardiomyocytes and easily penetrates the myocardium as fatty infiltrates, it can directly affect the myocardium through direct communication and secreted biomolecules [2]. In healthy humans fatty infiltrates of the myocardium are common: adipocytes constitute 1.2% of total cardiac cells [3]. Intramyocardial fat (IMF) volume correlates with the amount of EAT [4]. Moreover, EAT unlike other cardiac fat depots (peri- and paracardial fat) is supplied by branches of the coronary arteries and since both EAT and the myocardium share the same microcirculation, a direct cross-talk between the fat and the myocardium can occur.

EAT covers 80% of the surface of the heart and constitutes 20% of its total weight [5]. It is located mainly around epicardial blood vessels, in the atrioventricular and interventricular grooves with little fat present posteriorly [6] (Fig. 1). EAT amount over both ventricles is similar. EAT volume correlates with visceral adipose tissue (VAT) volume, but not with subcutaneous adipose tissue (SAT) and is also related to body mass index (BMI) and waist circumference [7].

EAT adipocytes are smaller than VAT and SAT adipocytes [8]. Moreover, while EAT expansion with progression of obesity occurs mainly through hyperplasia (addition of new adipocytes), hypertrophy (increased cell dimensions) of individual adipocytes is the dominant manner of adipose tissue expansion in visceral and subcutaneous fat depots [8–10], hence EAT adipocyte size does not increase with increased EAT volume or this increase is only minimal [11].

EAT hosts cardiac neurons and ganglionated plexuses, containing adrenergic and cholinergic neurons, both in the atria and ventricles, especially around large blood vessels [12, 13]. They create a network of interconnected fibers traveling along coronary arteries. This autonomic system innervates both EAT and the myocardium. EAT safeguards this cardiac autonomic nervous system against mechanical forces during cardiac contraction and, due to high expression of nerve growth factor-β (NGF-β), may provide a neuroprotective function.

EAT offers mechanical protection for the whole heart and especially for the coronary arteries and autonomic nerves. The rate of fatty acid absorption and release by EAT is approximately twice that of the pericardial depots, while glucose utilization is lower [14]. In view of proximity between EAT and cardiomyocytes, EAT may act as a local energy supply for adjacent myocardium, but also be a buffer against toxic levels of free fatty acids [14]. EAT could also protect the heart from hypothermia. In fact, polar bears present large amounts of cardiac fat that can be used to store and supply energy to the myocardium during hibernation [15].

EAT produces vast array of biomolecules that can affect both local coronary arteries and adjacent myocardium. They include adiponectin, adrenomedullin, omentin, leptin, resistin, interleukins, activin A. Secretory profile of EAT is different from that of other lipid depots [16]. Moreover, EAT secretome is affected by such risk factors as hyperglycemia or high free fatty acids [17] and undergoes significant changes in cardiovascular disease [16, 18]. EAT secretome affects both cardiomyocyte [16, 19] and fibroblast [20] function.

Pericoronary EAT (PCAT) located around large epicardial coronary arteries is a specific subsection of EAT [21] since it can interact bidirectionally with the vessel wall [22]. On the one way, PCAT is influenced by the vessel wall, but on the other it can exert effects on the adjacent arteries. Especially adiponectin produced and released by PCAT adipocytes was shown to be crucial for the normal structure and function of coronary arteries [23, 24]. It not only stimulates endothelial nitric oxide synthase (eNOS) to produce nitric oxide (NO), but also increases tetrahydrobiopterin availability, preventing eNOS decoupling [23]; moreover adiponectin inhibits vascular NADPH oxidase, promoting NO over superoxide radical [24]. Adiponectin downregulation is a hallmark of unhealthy, inflamed EAT and thus adiponectin deficiency may be a trigger of local pro-atherogenic and pro-inflammatory milieu, perpetuating the vicious circle of local inflammation.

Role of IMF is uncertain: intramyocardial adipocytes may secrete adipocytokines (such as adiponectin) that have beneficial effects on the survival of cardiomyocytes and vascular function [25], however excessive IMF has been associated with myocardial lipotoxicity, arrhythmogenic right ventricular cardiomyopathy, slow conduction, ventricular and atrial arrhythmias [26–28].

## Imaging methods of EAT

EAT can be assessed using various modalities, such as echocardiography, cardiac magnetic resonance imaging (cMRI), and cardiac computed tomography (cCT). Each of them offers unique benefits, but also has its drawbacks.

Transthoracic echocardiography identifies EAT as a hypoechoic space between the outer wall of myocardium and the visceral layer of pericardium. It usually measures only EAT thickness in a single location, which is a major limitation of this method. cMRI enables easy assessment and volumetric quantification of EAT, but is expensive, not widely available and conventionally does not provide information on EAT quality. However, a recent paper [29] demonstrated that cMRI may provide data on EAT composition, i.e. saturated/monounsaturated/polyunsaturated FFA ratio in EAT, offering complimentary information to that provided by CT imaging. Although this technique could become a valuable research and prognostic tool, its development has only just begun and we only have limited data regarding its usefulness.

**Fig. 2:**
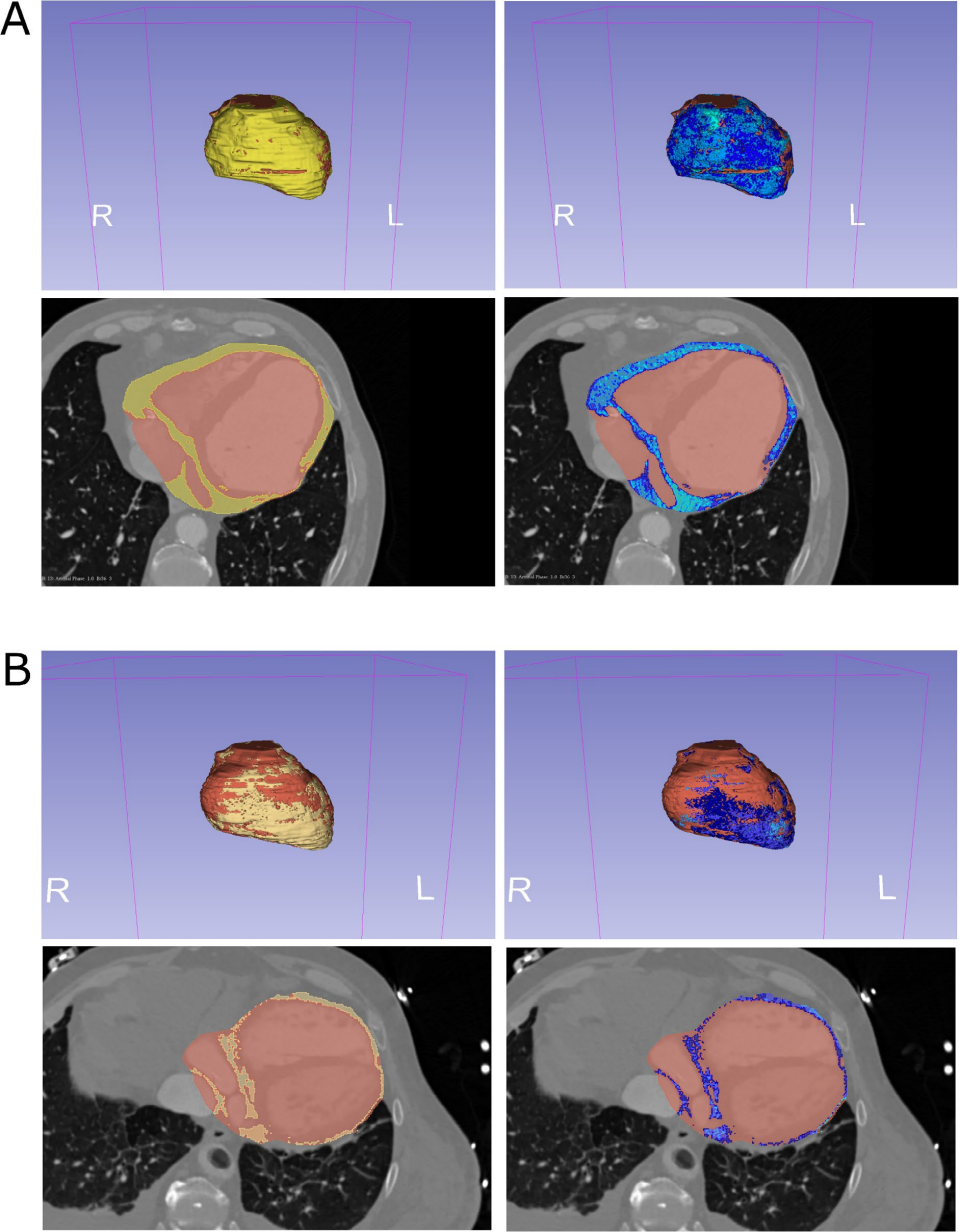
Computed tomography imaging of epicardial fat. Epicardial adipose tissue (EAT) volume and density analysis in contrast-enhanced computed tomography (CT) scan of the chest using open-source software 3D Slicer (https://www.slicer.org/) (our unpublished data). Left panels - red color indicates pixels within the space limited by the visceral layer of the pericardium (traced manually), whereas yellow color indicates EAT (detected using density threshold from -30 HU to -180 HU). Right panels - EAT density gradients: light blue - the lowest (-180 to -120), through blue (-120 to -90) to dark blue (-90 to -30). Upper panels - 3D reconstructions, lower panels - a single transverse slice. **[A]** Patient 1 with a thick layer of EAT, with low EAT density. **[B]** Patient 2 with a thin layer of EAT and high EAT density

Finally, cCT provides excellent EAT visualization, enabling both volumetric and thickness measurements (Fig. 2). Usually initially the visceral layer of the pericardium and the myocardial border are manually traced and subsequently all voxels located between these two lines that exhibit density lower than water (usually in the range of -30 to -180 Hounsfield units [HU] are counted as EAT (Fig. 2). Finally three-dimensional rendering produces a volumetric reconstruction of EAT (Fig. 2) so that total EAT volume and its specific subsections (e.g. ventricular, atrial, pericoronary) can be calculated.

cCT is unique since it also provides information on EAT density, so called fat attenuation index (FAI) [22], which reflects EAT quality. In particular coronary CT angiography (CCTA) can provide information on EAT located around main coronary arteries. These images can be analyzed using machine learning and artificial intelligence-based approaches, providing useful prognostic and diagnostic information [22, 30–32] (see “Coronary perivascular EAT density and vessel inflammation - who inflames who”) section.

## Coronary perivascular EAT density and vessel inflammation - who inflames who

**Fig. 3:**
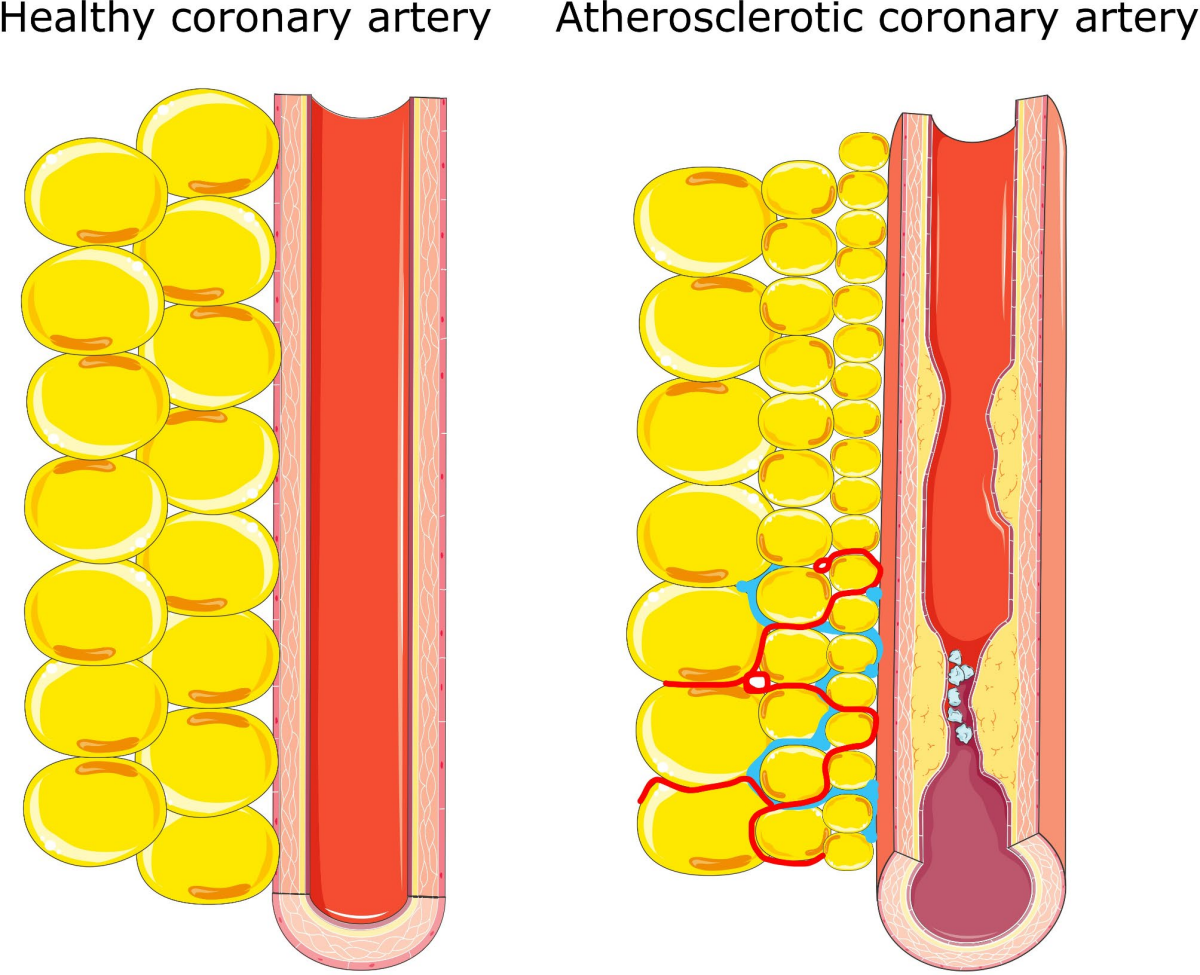
Adipocyte phenotype of pericoronary fat (PCAT) in normal versus atherosclerotic coronary arteries. In healthy arteries (left panel), PCAT adipocytes exhibit a typical and uniform size with ample intracellular lipid accumulation. In contrast, coronary inflammation associated with atherosclerotic plaques, especially unstable ones (right panel) alters this phenotype by reducing adipocyte size and lipid content due to inhibited maturation of preadipocytes into adult adipocytes in the area immediately adjacent to the vessel wall. This effect diminishes with increasing distance from the vessel. While these changes seem reversible, chronic vascular inflammation and atherosclerotic disease cause additional, irreversible changes in PCAT, including increased extracellular fibrosis (marked in blue) and microvascular remodeling (increased vascularity marked in red)

Human studies indicate that PCAT density, reflected by FAI, is particularly increased (by as much as 8-15 HU) around coronary vessel segments characterized by unstable plaques (ruptured plaques [22, 33] and soft non-calcified plaques [34]), moderately increased (by 4-8 HU) around significant (> 50% or fractional flow reserve [FFR] < 0.8) atherosclerotic plaques ([33–38]) versus healthy vessel segments PCAT that exhibits comparable FAI to the other subsections of EAT, such as ventricular or atrial EAT (Table 1). Since the increased FAI is limited to PCAT around particular segments of coronary vessels rather than whole vessels or whole coronary tree, this strongly suggests that it results from local interaction of PCAT with the affected blood vessel. Of note, a mild FAI and adipocyte size gradient is present even around healthy coronary arteries and this gradient is increased around the affected coronaries (Fig. 3). Surprisingly, this effect persists over up to 20 mm distance from the vessel wall [22], which is much more than with PAAT (up to 4 mm) [39], though in both cases the most pronounced changes can be seen within 1 mm from the myocardial border. This suggests that region of interest for EAT imaging should be adequately selected to visualize these relations.

**Table 1:** Clinical studies investigation association of epicardial tissue density with clinical outcomes

Study	N	Population	Outcome
Perivascular EAT			
[31]	3912	Subjects undergoing diagnostic CCTA	High PCAT FAI around proximal right coronary artery and LAD (≥–70.1 HU) was associated with increased risk of all-cause mortality (HR = 2.55) and cardiac mortality (HR = 9.04)
[22]	277	267 patients with stable CAD, 10 patients with acute MI	PCAT FAI predicted atherosclerotic plaque burden, exhibited density gradient (density reduced with distance from the vessel), and was especially high around ruptured plaques (MI within the past 72 h)
[41]	125	Patients with chronic CAD who underwent PCI	Patients who developed periprocedural myocardial injury had higher PCAT FAI (N = 50, -75.9 HU) than those without the injury (N = 75, -84.9 HU). Higher FAI predicted microvascular dysfunction
[35]	61	Patients with chronic CAD, a total of 77 coronary stenoses. 37 arteries with FFR > 0.8, and 40 arteries with FFR < 0.8	PCAT FAI for arteries with FRR < 0.8 had higher FAI (-66 HU) than for those with z FRR > 0.8 (-75 HU). No correlation with PCAT volume was found
[40]	364	Patients with suspected CAD	Patients with CFR < 2.5 (N = 206, -75.5 HU) had higher PCAT FAI than patients with CFR > 2.5 (N = 158, -77.1 HU), also in a group without obstructive lesions
[34]	210	Subjects with suspected CAD, undergoing CCTA: Group 1 subjects with normal CCTA, Group 2: non-obstructive atherosclerosis and Group 3: obstructive atherosclerosis	PCAT FAI was higher adjacent to the lesion (− 80 HU) compared to the normal segment (− 109 HU). higher in the obstructive group (− 59 HU) compared to the non-obstructive group (− 102 HU), but lower in calcified (− 90 HU) compared to non-calcified (− 75 HU) lesions
[65]	99	33 patients with obstructive atherosclerosis, 33 patients with non-obstructive atherosclerosis, 33 patients with normal coronary arteries	PCAT FAI around the most significant stenosis was highest (-127 HU) in the obstructive > non-obstructive (-162 HU) = normal group (-168 HU). No correlation between PCAT volume and stenoses was found
[33]	180	60 patients with acute (< 48 h) MI, 60 patients with stable CAD and 60 HC	PCAT FAI around proximal RCA was highest in patients with acute MI (-82 HU), then in patients with stable CAD (-91 HU) and lowest in HC (-96 HU). Total EAT FAI correlated with PCAT FAI in HC and CAD patients, but not in those with MI
[36]	187	Stable CAD patients with intermediate stenosis of LAD, undergoing FFR measurements	PCAT FAI around LAD > 71 HU predicted low FFR
[42]	212	Patients with MINOCA or Tako-Tsubo syndrome (n = 106) and HC (n = 106)	Mean PCAT FAI of 3 main coronary arteries (no difference between individual arteries) was -68 HU in patients vs. -78 HU in HC With cut-off value for inflammation − 70.83 HU, 55% of MINOCA/Tako-Tsubo subjects had inflamed coronary arteries, while coronary inflammation was found in only 5% of controls
[37]	138	Patients with angina undergoing FFR measurement	FFR < 0.75 PCAT FAI (-70) vs. 0.75 ≤ FFR ≤ 0.8 (-74), FFR > 0.8 (-79)
[78]	769	Inpatients infected with COVID-19	Hospital mortality was associated with PCAT FAI, but not with atherosclerotic plaque burden
[38]	246	Patients with suspected CAD undergoing FFR assessment	Patients with FFR < 0.8 had higher PCAT FAI at the lesion site (-71 HU) than patients with FFR > 0.8 (-76 HU); however no differences were found in PCAT FAI at normal sites, in total EAT volume or FAI
[79]	199	Patients with intermediate risk of CAD, undergoing CCTA	Lesion based PCAT FAI does not correlate with high-risk plaque features or plasma CRP
Periatrial EAT			
[53]	160	80 patients undergoing AF ablation and 80 HC	Mean EAT-LA FAI was higher in AF versus non-AF participants
[57]	732	Patients with nonvalvular AF who underwent cardiac CTA before PVI	Total EAT volume in patients with AF recurrence was higher, while total EAT FAI was lower (-69.1 HU vs -67.5 HU)
[56]	389	Patients undergoing PAF ablation	Neither total EAT volume, total EAT FAI, nor EAT-LA FAI was significantly associated with AF recurrence after PVI
[54]	460	Patients with AF referred for first AF catheter ablation	Patients with higher FAI (≥–96.4 HU) of the posterior LA EAT showed higher AF recurrence rates compared with patients with lower FAI (< − 96.4 HU)
[55]	73	43 consecutive patients who underwent catheter ablation for AF and 30 control patients	All measurements of EAT FAI were higher in patients with AF than in HC (entire atrium, right atrium, LA). Among patients with AF who underwent ablation, all EAT FAI measurements were higher in patients with recurrent AF than in those without. All atrial EAT FAI values predicted recurrent AF (EAT right atrium FAI: HR, 1.2; EAT-LA FAI: HR, 1.1)
Total EAT			
[62]	467	Patients with suspected ACS, undergoing CCTA	Patients with high-risk plaques had lower total EAT FAI (-88.1 HU) and higher total EAT volume (59 cm^3^) than those with low risk plaques (-86.9 HU) and (49 cm^3^)
[61]	1912	Asymptomatic subjects with cardiovascular risk factors who underwent CCTA. Over 14.5 years of follow-up 76 of them developed MI or cardiac death	Patients with cardiac event had lower total EAT FAI (-76.1 HU) vs. (-73.7 HU) and higher total EAT volume
[63]	456	Asymptomatic subjects with cardiovascular risk factors who underwent CCTA	No difference in total EAT FAI between patients with CCS = 0 (-75.7 HU) and CCS 1–100 (-75.8 HU), but lower in patients with CCS > 100 (-77.9 HU). Patients with CCS > 100 had larger total EAT volume. High total EAT volume and low total EAT density were associated with plasma inflammatory markers
[64]	255	Patients with atypical chest pain	Low total EAT FAI (-86 HU vs. -84 HU), but not total EAT volume predicted obstructive CAD
[80]	609	Patients with low to moderate risk of CAD	Patients with positive coronary artery calcification had lower total EAT FAI (-88 HU vs. -87 HU) and higher total EAT volume than those without
[66]	140	Subjects with cardiovascular risk factors	Patients with increased fasting glucose had by 1.1 HU lower total EAT FAI, diabetics by 3.2 HU lower and overweight/obese patients by 3.0 HU lower total EAT FAI. Total EAT FAI negatively correlated with total EAT volume
[67]	1948	Subjects with impaired fasting glucose, impaired glucose tolerance, diabetes and HC	EAT FAI: type 2 diabetes -72 HU, impaired glucose tolerance -71 HU, impaired fasting glucose and normal glucose -69 HU
[81]	178	Healthy subjects undergoing CCTA	Larger total EAT volume and higher total EAT FAI correlated with increased left ventricular mass, thickness and volumes. The highest parameters were observed in patients with above-median total EAT volume and FAI
[69]	154	Patients with HF with preserved ejection fraction	Total EAT FAI inversely correlates with total EAT volume; low total EAT FAI was associated with obesity, high fasting plasma glucose, and insulin resistance & predicted HF hospitalizations and deaths

ACS, acute coronary syndrome; AF, atrial fibrillation; CAD, coronary artery disease; CCTA, coronary computed tomography angiography; EAT, epicardial adipose tissue; FAI, fat attenuation index; FFR, fractional flow reserve; HC, healthy control; HF, heart failure; HR, hazard ratio; HU, Hounsfield units; LA, left atrium; LAD, left anterior descending coronary artery; MI, myocardial infarction; MINOCA, myocardial infarction with non-obstructive coronary arteries; N, number of patients in the study group; PAF, paroxysmal atrial fibrillation; PCAT, pericoronary adipose tissue; PVI, pulmonary vein isolation

Moreover, this increase of PCAT FAI is also encountered around coronary arteries without obstructive plaques that exhibit reduced coronary flow reserve in stable coronary artery disease (CAD) patients [40] or microvascular dysfunction in patients undergoing percutaneous coronary interventions [41] as well as in patients with intermediate lesions [36] or with myocardial infarction with non-obstructive coronary arteries (MINOCA) or Tako-Tsubo syndrome [42] This suggests that PCAT may not only be a recipient of signals coming from the vessel wall, but also actively affect the territory supplied by the adjacent artery, presumably by the release of its mediators that penetrate into the lumen of coronary arteries.

An elegant study by Antonopoulos et al. [22] demonstrated that coronary vascular inflammation, in particular pro-inflammatory cytokines interleukin-6 (IL-6), tumor necrosis factor–α (TNF-α) and interferon-γ (IFN-γ) released by the coronary artery wall inhibit maturation of preadipocytes to adult adipocytes in the close vicinity of the vessel and that this effect diminishes with the distance from the vessel wall. Since this maturation involves expression of enzymes responsible for triglyceride accumulation, smaller, lipid poor adipocytes predominate in the close vicinity of inflamed arteries and this effect decreases with the distance from the inflamed artery (Fig. 3). Lipids generally exhibit low density and poorly attenuate X-rays versus water containing tissues. Thus large, lipid rich adipocytes that predominate in healthy EAT exhibit lower X-ray attenuation and low FAI compared to small, lipid poor adipocytes, abundant in the close proximity of inflamed arteries. Therefore, increased FAI is a sensitive index of coronary inflammation [22]. Other studies also confirm that inflammation of PCAT is associated with increased FAI [43].

As shown above, this index not only indicates local vascular inflammation and reflects plaque nature/stability, but also predicts adverse cardiac events independently of other known risk factors [31]. Its predictive power is reduced in patients who started therapy that potentially modifies EAT inflammation (statin, aspirin), which supports the inflammation-related origin of acute coronary events and the fact that FAI reflects dynamic, modifiable changes in EAT composition [31].

Recently an artificial intelligence powered model, based on FAI and coronary plaque metrics and clinical factors was shown to provide better cardiovascular risk assessment than traditional models [44].

While acute PCAT inflammation is reflected by increased concentration of pro-inflammatory cytokines and small adipocyte size and can be captured by FAI, chronic PCAT inflammation is characterized by increased fibrosis and angiogenesis that are highly heterogeneous and result in increased tissue heterogeneity [45]. Indeed, recent advances in imaging of coronary perivascular EAT demonstrate that its radiomic profile may provide additional information on adverse EAT remodeling beyond inflammation, i.e., detect signs of fibrosis and increased vascularity [32], not captured by FAI.

Systemic factors also can affect PCAT. Indeed, animal studies suggest that high fat feeding upregulates pro-inflammatory gene expression in perivascular adipocytes [46]. Diabetic patients with poor glycemic control may have higher coronary local inflammation as detected by pericoronary FAI surrounding the three major coronary arteries [47]. In this context metformin was associated with lower PCAT FAI (by approx. 2 HU) [48], except in the obese subjects. Additionally, PCAT FAI was correlated with cardiovascular events in diabetic patients [49].

## Periatrial fat density and its association with atrial fibrillation

Both atria are surrounded by a specific periatrial EAT (PAAT) depot that exhibits unique transcriptomic signature [50]. Total EAT volume [51], but especially PAAT volume [52], is associated with incidence of atrial fibrillation (AF) and this association is independent from BMI. Most [53–55], but not all [56] studies suggest that increased PAAT FAI predicts AF attacks, AF recurrence following catheter ablation and AF persistence. The opposite relation was found for total EAT, i.e. reduced total EAT FAI predicts AF [57]. This suggests that local rather than systemic factors are responsible for the biology of PAAT.

PAAT potentially contributes to the development of AF through several mechanisms, including fatty atrial infiltration, inflammation, production and release of reactive oxygen species and autonomic nervous system dysfunction. Inflammation has been proposed as one of the main pathogenetic mechanisms linking EAT and AF [58].

Recently Ishii et al. [39] showed that PAAT is composed of two distinct adipocyte populations: marginal adipocytes adjacent to the atrial myocardium and central adipocytes located away from both the myocardium and epicardium. Both adipocyte populations exhibited hypertrophy related to BMI and total EAT volume and related PAAT FAI also was reduced. However, in patients with paroxysmal, and especially permanent AF the marginal adipocytes were smaller, this layer underwent severe fibrotic remodeling and the expression of pro-inflammatory cytokines was increased. Moreover, this was associated with increased atrial fibrosis, which could be a direct link between PAAT and AF. Thus, in PAAT increased FAI may result not only from reduced adipocyte size, but also from severe fibrosis. Of note, the best predictor of AF was not FAI, but its gradient from the PAAT-myocardium border to the central area: high gradient was associated with increased AF risk. Thus FAI, both its average value and its gradient could be used to assess the risk of AF occurrence/recurrence [58]. Similarly, large PAAT FAI dispersion was shown to predict AF recurrence after pulmonary vein isolation [59].

## Periventricular epicardial fat - a marker of systemic metabolism or an inducer in itself

Since periventricular EAT (PVentAT) accounts for approximately 2/3 of the whole EAT (our unpublished data), analysis of total EAT largely reflects the characteristics of PVentAT.

Surprisingly, low total EAT FAI is associated with increased mortality [60] and with adverse cardiovascular events [61]in apparently healthy subjects. Moreover, mean total EAT FAI is lower in patients with high risk plaque characteristics [62], high plaque burden [63], and obstructive CAD [64], though not all studies support this association [64, 65]. Increased fasting glucose and impaired glucose tolerance are associated with slightly reduced EAT FAI (by 1–2 HU), while overt diabetes with even more reduced EAT FAI (by 3–4 HU) [66–68]. Obesity is also associated with reduced total EAT FAI [66–68]. In heart failure with preserved ejection fraction, total EAT FAI is reduced and associated with a higher incidence of heart failure readmissions and composite endpoint [69].

How can we explain this apparent paradox? How can low total EAT density have exactly the opposite prognostic effect in various populations, from apparently healthy subjects, through obese and diabetic patients, to heart failure with preserved ejection fraction (HFpEF) and CAD patients, than local PCAT and PAAT density?

Total EAT FAI is negatively correlated with total EAT volume. The association is weak and the differences between the study groups usually are within 1–2 HU, though they are present in all populations, such as in healthy, diabetic, CAD and HFpEF subjects [66–68, 70]. Even longitudinal changes of total EAT FAI and volume go in opposite directions [71]. On the other hand, total EAT volume is associated with systemic metabolic factors, such as visceral obesity, waist circumference and BMI. Therefore, low EAT FAI may just be a marker of systemic metabolic factors that have their own adverse cardiovascular effects.

**Fig. 4:**
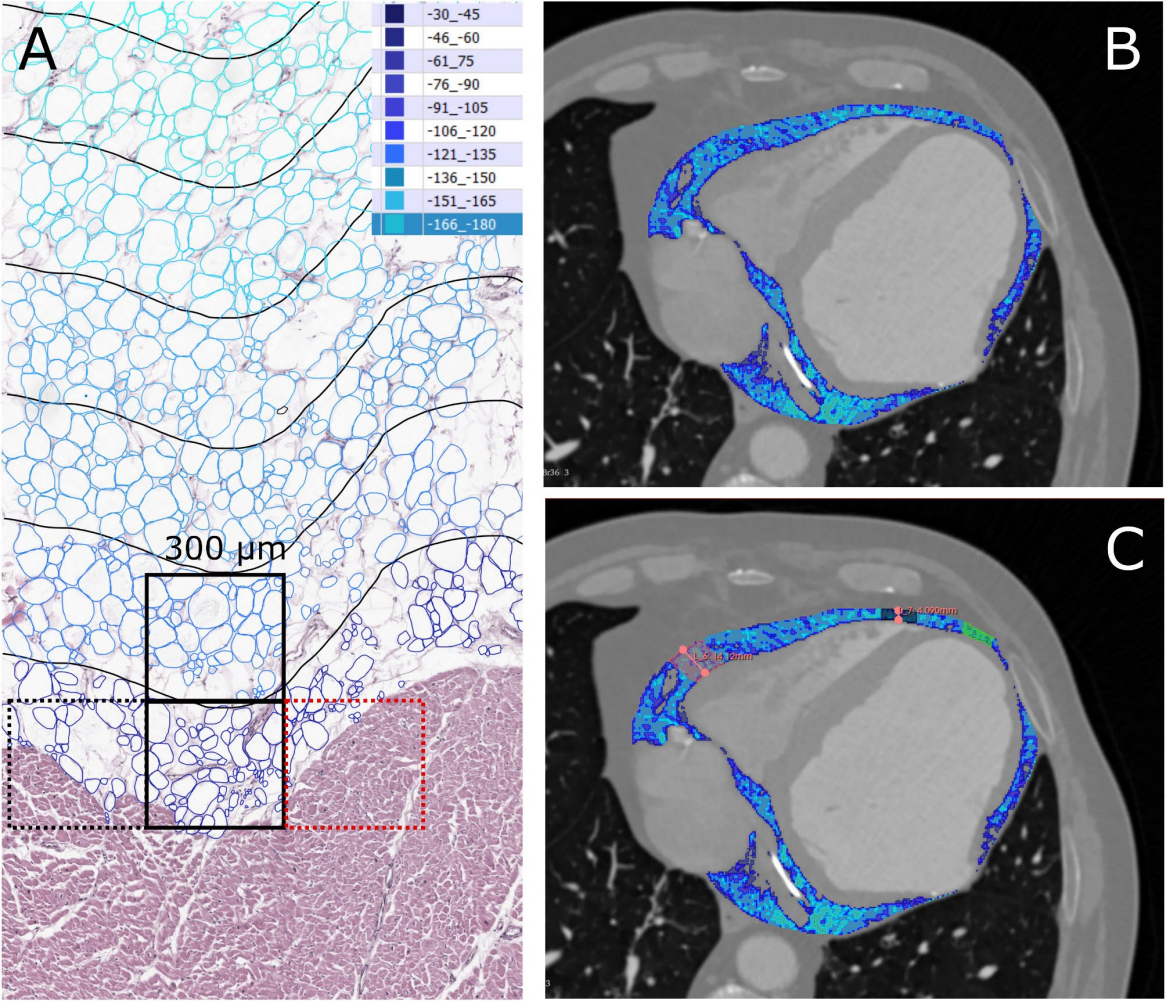
Challenges in analyzing the density of epicardial adipose tissue. A. Hematoxylin and eosin stained section of healthy human epicardial adipose tissue adhering to the myocardium. Individual adipocytes are marked with various shades of blue. The voxel size of conventional computed tomography (CT) scanners (300 µm in this example, shown as a black square) is significantly larger than the average diameter of an epicardial adipose tissue (EAT) adipocyte (~ 50 µm). Consequently, each voxel reflects the density of a group of cells, including both adipocytes and cardiomyocytes (see adjacent voxels shown in dashed black and red), especially in border regions such as the myocardium/EAT interface. This overlap can lead to an overestimation of EAT density. Black curves indicate consecutive 300 µm distances from the myocardium, corresponding to the areas of subsequent voxels in CT images. B, C. CT slices of the healthy human heart with the epicardial fat marked in shades of blue (legend included as inset to A). In cases with a thick EAT layer (marked in red), these border regions with potentially overestimated fat attenuation index (FAI) contribute less to the total EAT FAI compared to cases with a thinner EAT layer (marked in green). This is reflected by a gradual increase of EAT FAI with the decrease of EAT thickness in the same patient (our unpublished data)

**Fig. 5:**
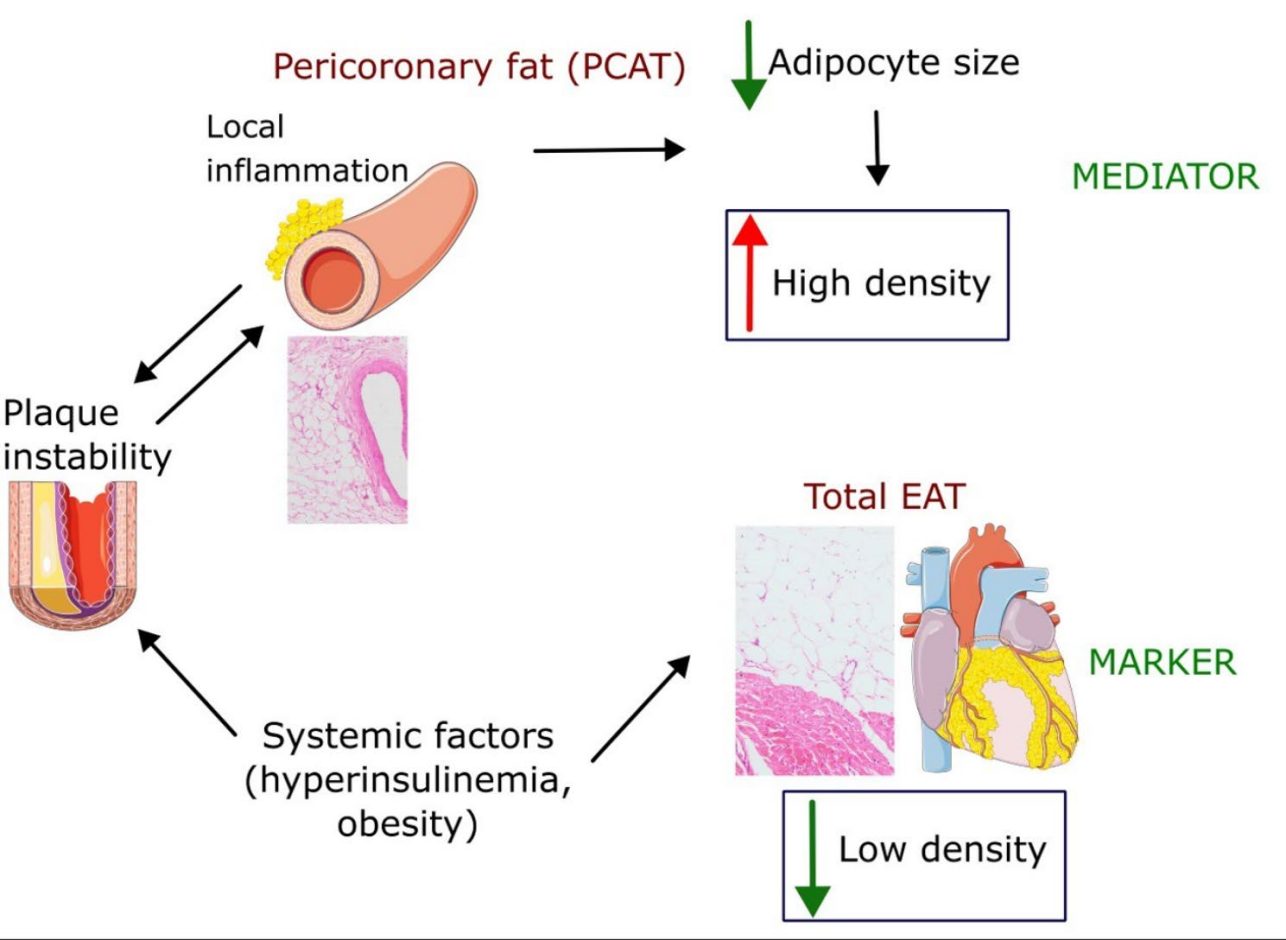
Different relation between pericoronary and total epicardial fat density and condition of a coronary artery. There is a bidirectional interaction between the pericoronary adipose tissue (PCAT) and the coronary artery (indicated by two oppositely directed arrows). PCAT inflammation results in (1) production of adipo-fibro-cytokines that may trigger development of atherosclerotic plaques, destabilize the plaques and affect microcirculation supplied by the coronary artery, and (2) reduction of adipocyte size that is reflected by increased adipose tissue density and increased fat attenuation index (FAI) in computed tomography imaging. On the other hand, total EAT density reflects action of systemic factors, such as hyperinsulinemia, obesity, that tend to reduce its density and FAI and independently affect the coronary arteries. Thus while high FAI of PCAT may reflect direct involvement of inflamed PCAT in coronary pathology (MEDIATOR), low FAI of total EAT may reflect action of systemic factors that exert their effects on coronary arteries that are not mediated by EAT (MARKER)

But why there is a reduced total EAT FAI in subjects with large total EAT volume? First, it could be an artifact related to inadequacy of imaging methods. The minimum diameter of voxels produced by conventional modern CT scanners (> 200 µm) is significantly larger than the average diameter of an EAT adipocyte (approximately 50 µm), so a single voxel reflects the density of a group of several dozen cells [9, 10]. Thus, in the border regions, such as the myocardium/EAT, a single voxel may represent an average of some cardiomyocytes and adipocytes, which would necessarily overestimate the EAT density (Fig. 4). In cases of thick EAT layer those border regions with potentially overestimated FAI contribute less to total EAT FAI than in cases of thin EAT layer. Second, low EAT FAI may reflect adipocyte hypertrophy and EAT expansion due to systemic factors (e.g., insulin resistance) associated with increased cardiovascular risk. Although EAT adipocyte size does not seem to increase much with progression of systemic obesity and EAT expansion (unlike in other fat depots) [8–10], some minor increases were found [11, 39] that could nevertheless account for small reduction of EAT FAI found to correlate with EAT expansion. Last but not least, adipocyte hypertrophy and hyperplasia could result in tissue hypoxia if rapid adipose tissue expansion exceeds vascular growth, triggering local inflammation that eventually might have adverse effects on the underlying myocardium, as in PCAT and PAAT (Fig. 5). However, this possibility seems unlikely since EAT inflammation should be associated with increased rather than reduced FAI. Finally, low EAT FAI could reflect a reduced brown to white adipose tissue ratio. Brown adipose tissue is characterized by “healthy” phenotype, small cells and high FAI as opposed to white adipose tissue with “unhealthy” phenotype, large cells and low FAI. However, brown adipocytes have distinct appearance and they are rarely found in human EAT, so their contribution to total EAT characteristics is most probably negligible [63].

**Fig. 6:**
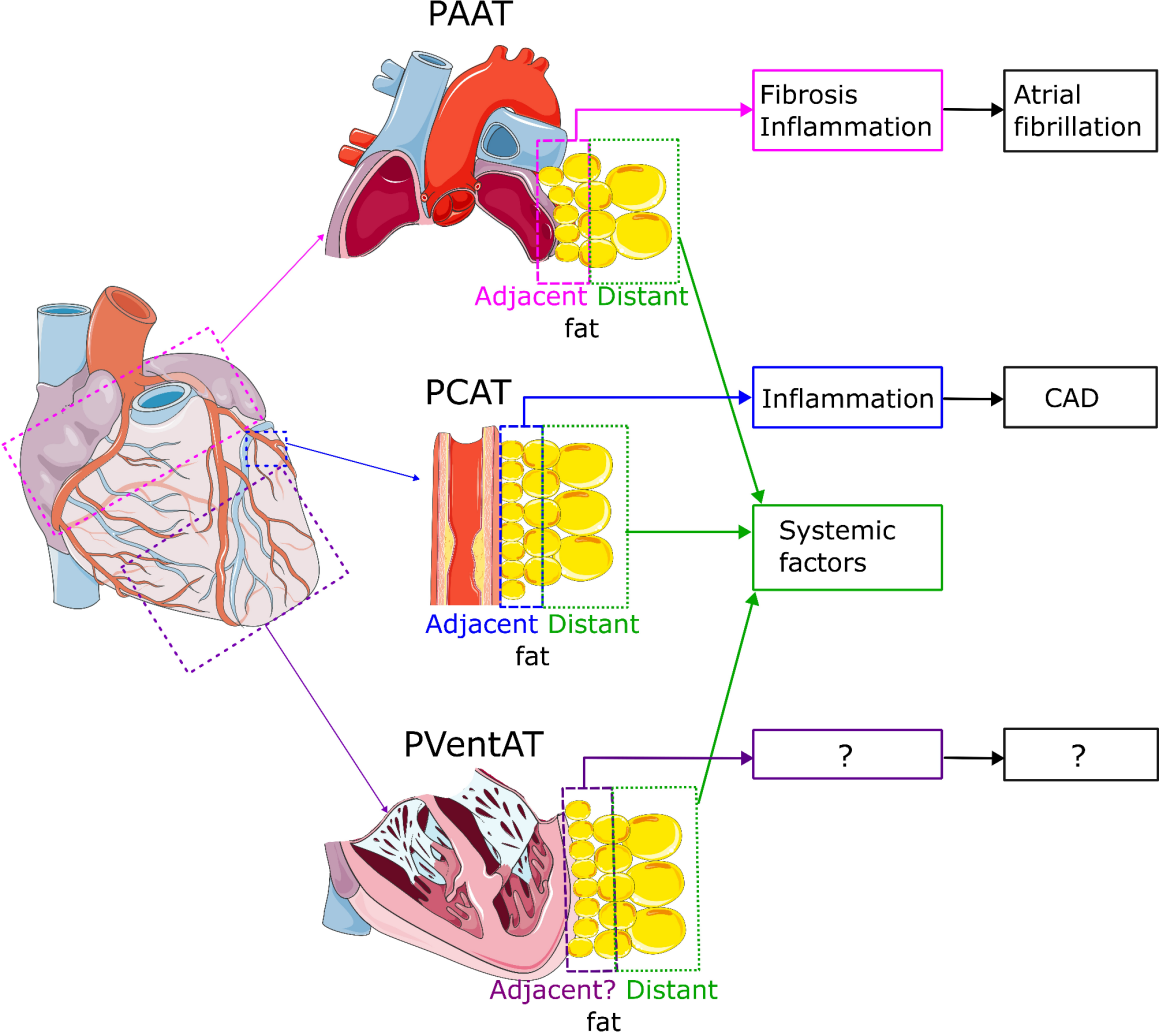
Different relevance of the epicardial adipose tissue located close (adjacent fat) and away (distant fat) from target tissues. Adjacent fat holds significant prognostic value due to its bidirectional interaction with the target tissue. Increased density of adjacent pericoronary adipose tissue (PCAT) is associated with coronary artery disease (CAD) and indicates inflammation. Elevated density of adjacent periatrial adipose tissue (PAAT) correlates with atrial fibrillation, reflecting both inflammation and fibrosis. It is unknown if the concept of adjacent epicardial adipose tissue applies to the periventricular adipose tissue (PVentAT). In contrast, distant fat has no immediate effect on the target tissue, but is a marker of systemic factors, such as obesity or hyperinsulinemia, that have their own detrimental cardiovascular effects probably not mediated by the epicardial fat

Of note, while EAT density and adipocyte size gradient have been demonstrated for PCAT and PAAT adjacent to diseased coronary arteries and atria, respectively, no such studies were performed for PVentAT and failing hearts. The question whether PVentAT interacts with ventricular myocardium, as the other EAT depots with their target tissues, warrants urgent investigation (Fig. 6).

## EAT distribution and pathophysiology of cardiovascular conditions

In healthy humans PAAT accounts for approximately 20% of total EAT, while LV and RV EAT each account for approximately 40% of it, while the amount of PCAT is negligible. Since RV myocardial mass is approximately 1/3 of LV weight, adipose tissue to myocardium ratio is 3 times higher for RV than for LV [72, 73]. Evidence indicates that this EAT distribution changes in various pathologies. PAAT to PVentAT is increased in patients with recurrent AF despite pulmonary vein isolation [74]. Total EAT volume is increased in HF [4, 75, 76], however while in HFpEF and heart failure with moderately reduced ejection fraction (HFmrEF) this increase is proportional to systemic markers of obesity, such as BMI and affects equally all EAT sites (PAAT, PVentAT), in heart failure with reduced ejection fraction (HFrEF) PVentAT is increased only around LV, but due to LV hypertrophy and dilation, EAT/myocardium ratio and PVentAT thickness over LV are actually decreased. On the other hand, PCAT thickness changes proportionally to total EAT thickness [34, 77]. The consequences of these differences is not completely understood.

## Future perspectives

Further studies are needed to demonstrate whether (1) there is a causal relation between EAT and cardiovascular disorders, or EAT is a mere indicator of pathology, e.g. inflammation, (2) EAT density can be modified using therapies to improve cardiovascular risk, (3) PVentAT exhibits similar relation with its underlying ventricular myocardium as PCAT and PAAT do with their cardiac tissues and finally (4) if cMRI-derived information on chemical composition of EAT can provide important research and prognostic information.

## Summary

There is a universal understanding that the attenuation of a specific EAT depot has an established pathophysiological and prognostic significance: PCAT becomes denser as it undergoes pro-inflammatory transformation and is a prognostic marker of both local events related to the vessel adhering to the inflamed fat as well as remote events related to microcirculation supplied by this artery. PAAT becomes denser as it undergoes pro-inflammatory and pro-fibrotic transformation, resulting in pro-fibrotic and structural remodeling of the atrium, increasing the risk of AF.

Limitation of these FAI changes to specific fat depots (PCAT, PAAT) and clear FAI gradient, with more dense EAT, reflecting its pathological transformation, adhering to the specific structure (coronary vessel wall and atrial myocardium) suggest that potential effects of the EAT on the underlying structures are restricted to these deepest layers, immediately adhering a vessel wall or the myocardium, so called adjacent fat (Fig. 6).

However periventricular fat, accounting for the majority of total EAT, remains a mystery. Surprisingly low total EAT FAI, corresponding to low EAT density, is associated with increased cardiovascular risk. The meaning of this observation is unknown, since this low EAT density could be a mere result of increased EAT volume, devoid of any significance on its own, and could reflect the action of systemic factors (such as insulin resistance) known to have adverse cardiovascular effects on their own or could signal future pro-inflammatory remodeling of such low density, hypertrophic EAT (Fig. 6). Moreover, while there is good evidence for bidirectional communication between PCAT and a specific artery as well as PAAT and a specific atrial myocardium, no such data exist as yet for PVentAT and the ventricular myocardium. Thus, there is an urgent need to test if a similar interaction can be found, for example in heart failure. This could be of clear clinical relevance, since such PVentAT could be both a diagnostic marker as well as a therapeutic target.

Moreover, the emerging data indicates that FAI can be an important diagnostic and prognostic tool in both CAD and AF. Future studies will demonstrate if it also could be used as a marker of efficacy of therapies and whether FAI PVentAT could indicate ventricular pathologies, such as heart failure.

## Data Availability

No datasets were generated or analysed during the current study.
